# Investigation of nitro–nitrito photoisomerization: crystal structures of *trans*-{2,2′-[ethane-1,2-diylbis(nitrilo­methyl­idyne)]diphenolato}(pyridine/4-methyl­pyridine)­nitro­cobalt(III)

**DOI:** 10.1107/S2056989018015487

**Published:** 2018-11-09

**Authors:** Shigeru Ohba, Masanobu Tsuchimoto, Naoki Yamada

**Affiliations:** a Research and Education Center for Natural Sciences, Keio University, 4-1-1 Hiyoshi, Kohoku-ku, Yokohama 223-8521, Japan; b Department of Chemistry, Chiba Institute of Technology, Shibazono 2-1-1, Narashino, Chiba 275-0023, Japan; c Department of Chemistry, Faculty of Science and Technology, Keio University, Hiyoshi 3-14-1, Kohoku-ku, Yokohama 223-8522, Japan

**Keywords:** crystal structure, nitro-nitrito photo-isomerization, reaction cavity

## Abstract

The reaction cavities of the nitro groups in the title compounds, *trans*-{2,2′-[ethane-1,2-diylbis(nitrilo­methyl­idyne)]diphenolato-κ^4^ *O*,*N*,*N*′,*O*′}(nitro-κ*N*)(pyridine-κ*N*)cobalt(III), [Co(C_16_H_14_N_2_O_2_)(NO_2_)(C_5_H_5_N)], (I), and *trans*-{2,2′-[ethane-1,2-diylbis(nitrilo­methyl­idyne)]diphenolato-κ^4^ *O*,*N*,*N*′,*O*′}(4-methyl­pyridine-κ*N*)(nitro-κ*N*)cobalt(III), [Co(C_16_H_14_N_2_O_2_)(NO_2_)(C_6_H_7_N)], (II), have been investigated to reveal that the inter­molecular C_Me_—H⋯O(nitro) contacts in (II) are unfeasible for the nitro–nitrito photochemical linkage isomerization process. In (I), there are two independent complexes showing similar conformations, and the central five-membered chelate ring of the tetra­dentate salen ligand adopts the same absolute configuration. This is the result of pseudo-spontaneous resolution, since the configuration of the five-membered chelate ring may frequently be reversed in solution. In the crystals of (I) and (II), the mol­ecules are linked into three-dimensional networks by C—H⋯O hydrogen bonds.

## Abstract 2

The crystal structures of the title compounds have been studied to clarify the characteristics of the NO_2_^−^ ligand as a C—H⋯O hydrogen-bond acceptor, in relation to the solid-state photochemical linkage isomerization.

## Chemical context   

The nitrite ion is an ambidentate ligand, which shows linkage isomerism. In a Co^III^ complex, nitro (N-bonded) coordination is thermodynamically more stable than the nitrito (O-bonded) form, but nitro-nitrito linkage isomerization may occur in the solid state by irradiation with visible or UV light (Balzani *et al.*, 1968 ▸; Coppens *et al.*, 2002 ▸). The crystal structures of *trans*-[Co(en)_2_(NO_2_)(NCS)]NCS (Ohba, Tsuchimoto & Kurachi, 2018 ▸) and *trans*-[Co(acac)_2_(NO_2_)(pyridine derivative)] (Ohba, Tsuchimoto & Miyazaki, 2018 ▸) indicated that a certain geometry of the inter­molecular N/C—H⋯O contacts restricts the photoisomerization. In the present study, we investigated another type of nitro­cobalt complex, *trans*-[Co(salen)(NO_2_)(*X*-py)], where H_2_salen is *N*,*N*′-bis­(salicyl­idene)-1,2-ethane­di­amine, and *X*-py is pyridine in (I) or 4-methyl­pyridine in (II).
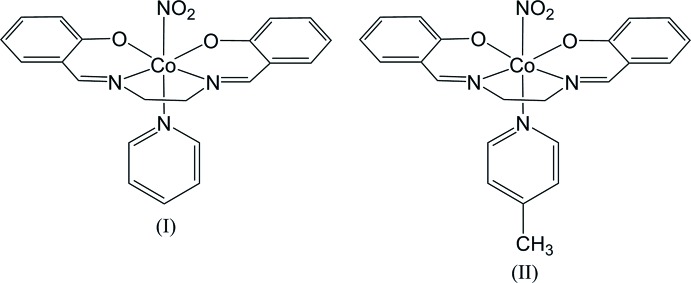


When the KBr disk of the py complex (I) was irradiated for 30 min with a Xe lamp, the colour changed from brown to reddish brown, and the IR spectrum showed an increase in intensity of the absorption peak in the region of 1040–1060 cm^−1^ (see figure in the supporting information), which corresponds to the symmetric N—O stretching mode of the nitrito form (Heyns & De Waal, 1989 ▸). The colour and IR spectrum reverted to those before irradiation on standing at room temperature for 2 h. On the other hand, the 4-Me-py complex (II) was photo-stable and did not show any change in the colour or IR spectrum upon irradiation. The crystal structures of (I) and (II) were determined to investigate the steric circumstances of the nitro ligand.

The photo-reactivities of nitro­cobalt complexes in the solid state depend not only on the steric conditions but also on the electronic effects of the co-existing ligands (Miyoshi *et al.*, 1983 ▸). The change of the IR spectrum of (I) upon irradiation was less apparent and it disappeared much more quickly after irradiation than that of *trans*-[Co(acac)_2_(NO_2_)(py)] (Ohba, Tsuchimoto& Miyazaki, 2018 ▸), indicating that salen^2−^ is not as suitable as acac^−^ for stabilization of the nitrito form.

## Structural commentary   

**Figure 1:**
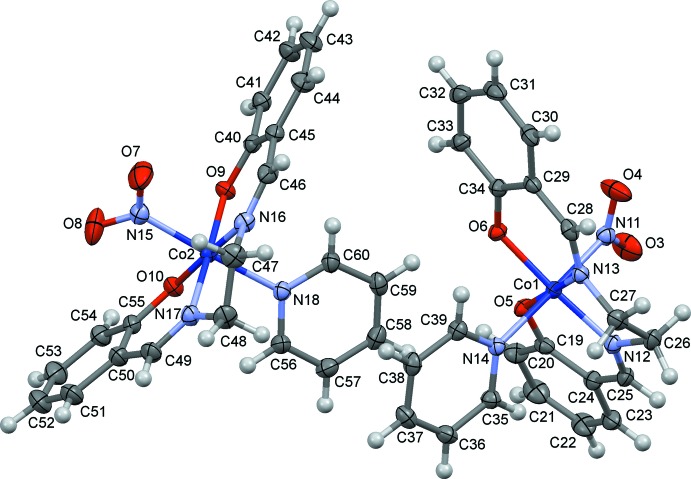
The mol­ecular structure of (I), showing displacement ellipsoids at the 30% probability level.

The mol­ecular structures of (I) and (II) are shown in Figs. 1 ▸ and 2 ▸, respectively. In (I), there are two independent complex mol­ecules, which have similar conformations, the five-membered chelate ring of salen being *gauche* with a λ form. The chirality of the crystal structure indicates that the crystals are pseudo-racemic conglomerates, because the configuration of the chelate ring may frequently switch from λ to δ, and *vice versa*, in solution. The Co—N(nitro) bond lengths are 1.944 (4) and 1.950 (3) Å in (I) and 1.916 (4) Å in (II). In each case, the coordination geometry around the Co atom is a distorted octa­hedron with the N(nitro) and N(py) atoms at the *trans* positions.

## Supra­molecular features   

**Figure 3:**
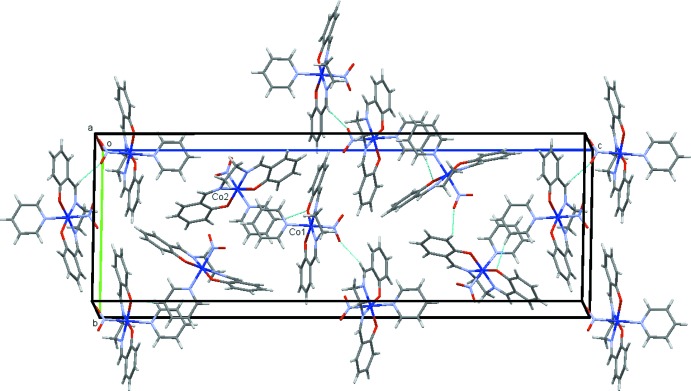
The crystal structure of (I), projected along *a*. The C—H⋯O hydrogen bonds are shown as blue dashed lines.

The crystal structures of (I) and (II) are shown in Figs. 3 ▸ and 4 ▸, respectively. In both (I) and (II), the mol­ecules are connected by C—H⋯O hydrogen bonds (Tables 1 ▸ and 2 ▸), forming a three-dimensional network. There are π–π inter­actions between the pyridine rings in (I) (see Figs. 1 ▸ and 3 ▸), the distance between the centroids being 3.82 (1) Å with a dihedral angle of 15.74 (8)°. The shortest contact between the rings is C39⋯C59 of 3.351 (6) Å.

**Figure 5:**
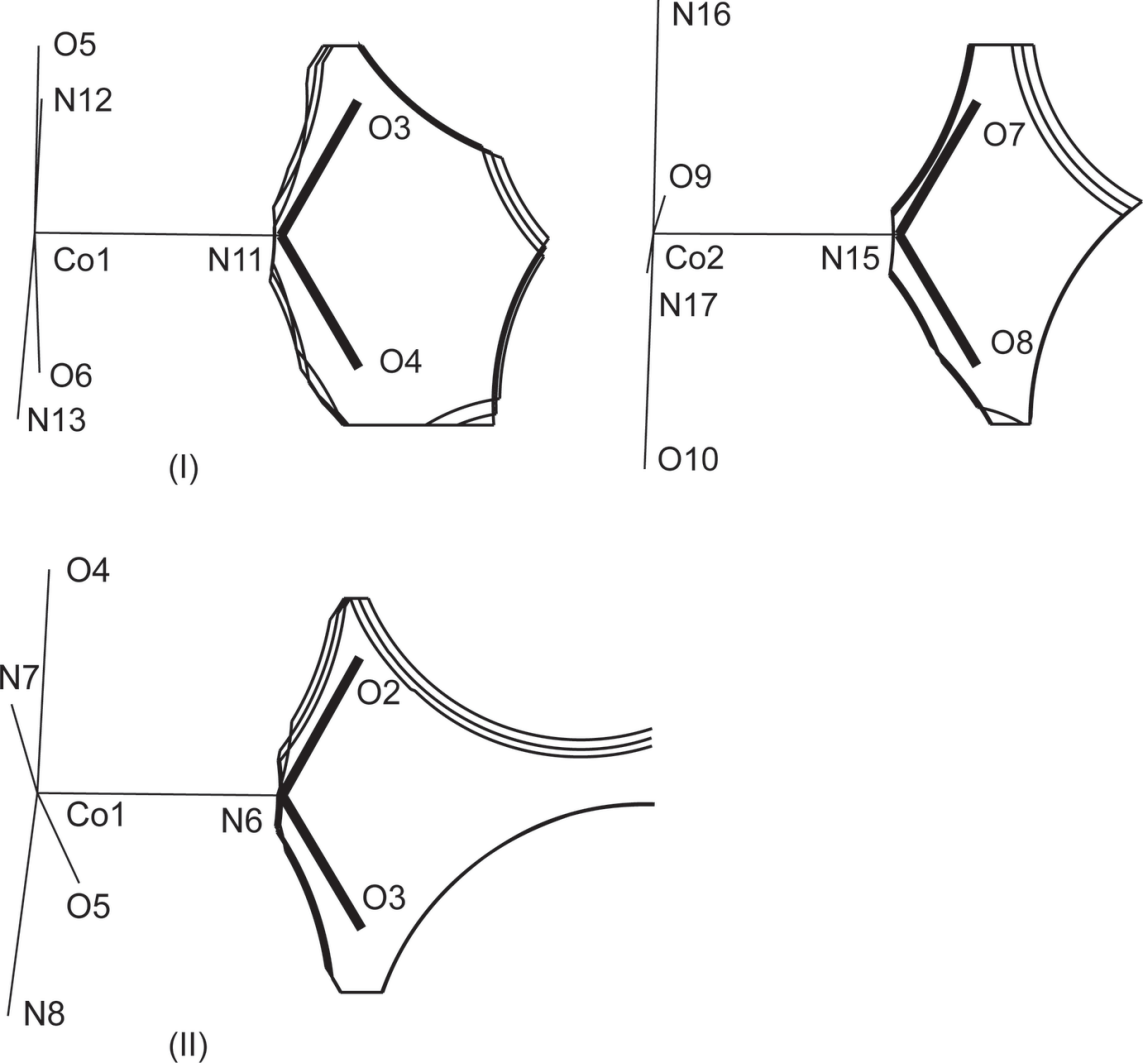
Comparison of the slices of the cavity around the nitro group within 0.1 Å from the plane in (I) and (II).

**Figure 6:**
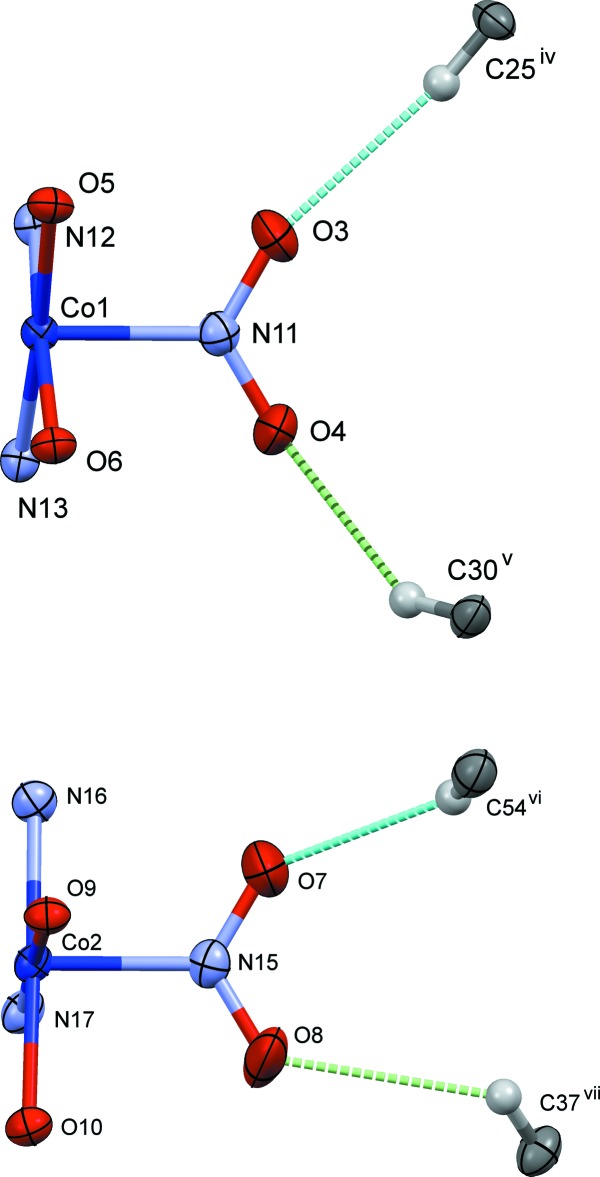
The steric circumstances of the nitro groups in (I). Only parts of the complex are shown for clarity. The C—H⋯O hydrogen bonds are shown as blue dashed lines. The green dashed lines indicate other O⋯H contacts shorter than 2.8 Å, O4⋯H30^v^ = 2.77 Å and O8⋯H37^vii^ = 2.66 Å. Symmetry codes: (i) *x* + 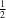, −*y* + 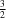, −*z* + 1; (ii) *x* + 1, *y*, *z*; (iii) −*x*, *y* + 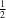, −*z* + 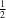; (iv) *x* − 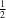, −*y* + 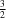, −*z* + 1; (v) *x* − 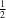, −*y* + 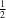, −*z* + 1; (vi) −*x*, *y* − 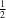, −*z* + 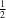;; (vii) −*x* + 1, *y* − 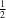, −*z* + 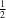.

**Figure 7:**
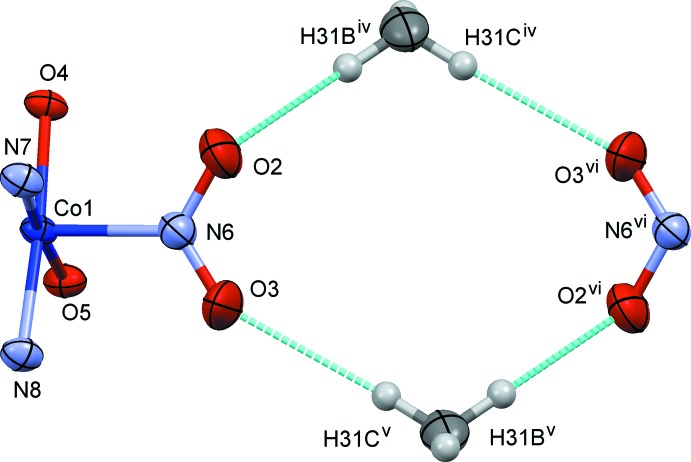
The steric circumstance of the nitro group in (II). Only parts of the complex are shown for clarity. The C—H⋯O hydrogen bonds are shown as blue dashed lines. Symmetry codes: (i) −*x* + 1, −*y* + 1, −*z* + 1; (ii) −*x* + 1, *y* − 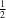, −*z* + 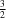; (iii) *x* + 1, −*y* + 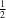, *z* + 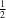; (iv) −*x* + 1, *y* + 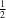, −*z* + 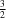; (v) *x* − 1, −*y* + 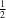, *z* − 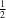; (vi) −*x*, 1 − *y*, 1 − *z*.

Slices of the reaction cavities around the NO_2_^−^ group near its plane in (I) and (II) are compared in Fig. 5 ▸, where the radii of neighboring atoms are assumed to be 1.0 Å greater than the corresponding van der Waals radii (Bondi, 1964 ▸) except for Co, its radius being set to 1.90 Å. The shape of the cavity in the nitro plane is mainly defined by the C—H⋯O(nitro) contacts, which are shown in Figs. 6 ▸ and 7 ▸. In (I), the cavity of O3—N11—O4 is wide enough to rotate in the original plane to achieve the N,O-bidentate transition state toward the nitrito form, in accord with the observed photo-activity of (I). In (II), the cavity of O2—N6—O3 has a tail, which is connected to that of the symmetry-related one, as seen in Fig. 7 ▸. These nitro groups are connected *via* C_Me_—H⋯O hydrogen bonds to form an 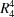
(12) ring, there being a narrow void around the center of the ring. The photo-stability of (II) suggests that the rotation of the NO_2_^−^ group in its plane will be blocked by the C—H⋯O hydrogen bonds. The steric condition of O7—N15—O8 in (I) is similar to that in (II), suggesting that the photoreaction in (I) mainly occurs at the Co1 complex site.

## Database survey   

There is no entry for *trans*-[Co(salen)(NO_2_)(*X*-py)] in the Cambridge Structural Database (CSD Version 5.39; Groom *et al.*, 2016 ▸), although the structures of related compounds have been published, for example *trans*-[Co(salen)(py)_2_][BPh_4_^−^] (Shi *et al.*, 1995 ▸) and *trans*-[Co(salen)(4-Cl-py)_2_][ClO_4_^−^]·CH_3_OH (Zhang, 2010 ▸).

## Synthesis and crystallization   

Cobalt(II) acetate tetra­hydrate, sodium nitrite, H_2_salen, and pyridine/4-methyl­pyridine (molar ratio 1:1:1:1) were reacted in methanol. Air was bubbled through the solution at 328 K for 1 h to precipitate the title compound. Brown needles of (I) and (II) were grown from a dimethyl sulfoxide solution and an *N, N′*-di­methyl­formamide solution, respectively, by diffusion of diethyl ether vapour.

## Refinement   

Crystal data, data collection and structure refinement details are summarized in Table 3 ▸. The H atoms bound to C were positioned geometrically, the methyl H atoms being introduced by an HFIX 137 command. They were refined as riding, with C—H = 0.93–0.97 Å, and *U*_iso_(H) = 1.2*U*_eq_(C) or 1.5*U*_eq_(C_Me_). (I): Since the *c* axis is longer than 40 Å, the overlapping of reflections was avoided in the intensity measurement by a longer sample-to-detector distance than the usual. (II): Six reflections showing poor agreement were omitted from the final refinement.

## Supplementary Material

Crystal structure: contains datablock(s) I, II, general. DOI: 10.1107/S2056989018015487/hb7784sup1.cif

Structure factors: contains datablock(s) I. DOI: 10.1107/S2056989018015487/hb7784Isup2.hkl

Supporting information file. DOI: 10.1107/S2056989018015487/hb7784Isup4.cdx

Structure factors: contains datablock(s) II. DOI: 10.1107/S2056989018015487/hb7784IIsup3.hkl

Supporting information file. DOI: 10.1107/S2056989018015487/hb7784IIsup5.cdx

The IR spectra of pyridine compound (I) before and after photoirradiation for 30 min by a 150 W Xe lamp to the KBr disk. DOI: 10.1107/S2056989018015487/hb7784sup6.tif

CCDC references: 1876726, 1876725

Additional supporting information:  crystallographic information; 3D view; checkCIF report

## Figures and Tables

**Figure 2:**
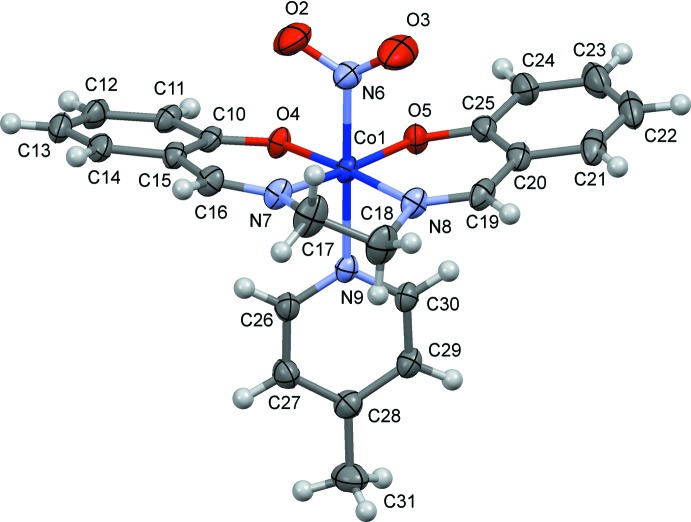
The mol­ecular structure of (II), showing displacement ellipsoids at the 30% probability level.

**Figure 4:**
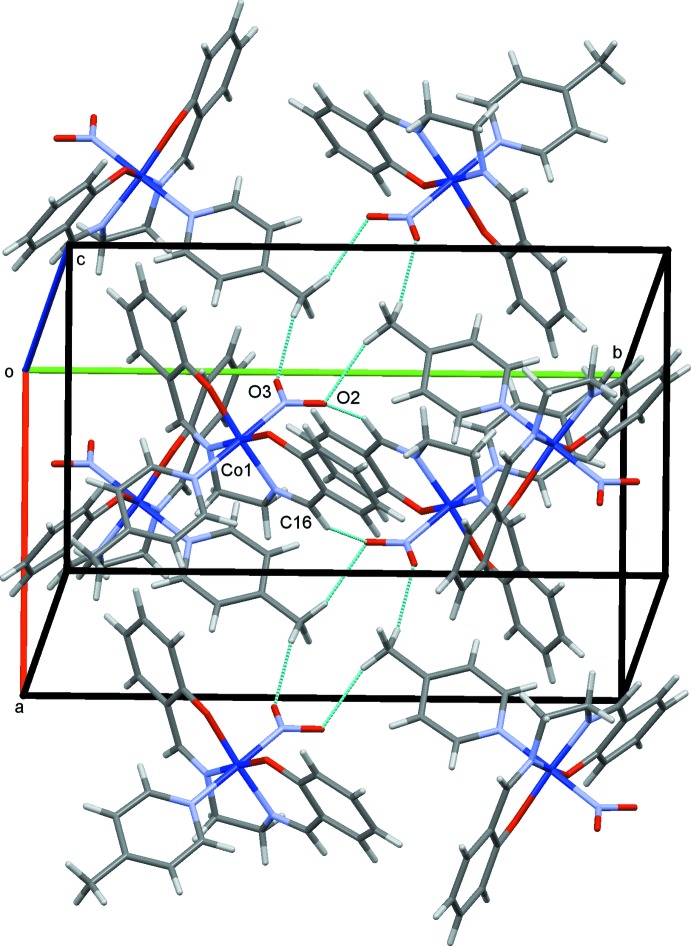
The crystal structure of (II), projected along *c*. The C—H⋯O hydrogen bonds are shown as blue dashed lines.

**Table 1:** Hydrogen-bond geometry (Å, °) for (I)

*D*—H⋯*A*	*D*—H	H⋯*A*	*D*⋯*A*	*D*—H⋯*A*
C25—H25⋯O3^i^	0.93	2.48	3.341 (5)	154
C38—H38⋯O9^ii^	0.93	2.39	3.280 (5)	160
C48—H48*B*⋯O8^ii^	0.97	2.48	3.285 (7)	140
C54—H54⋯O7^iii^	0.93	2.54	3.291 (7)	138
C59—H59⋯O6	0.93	2.38	3.213 (5)	149

Symmetry codes: (i) 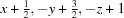; (ii) 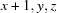; (iii) 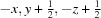.

**Table 2:** Hydrogen-bond geometry (Å, °) for (II)

*D*—H⋯*A*	*D*—H	H⋯*A*	*D*⋯*A*	*D*—H⋯*A*
C16—H16⋯O2^i^	0.93	2.58	3.358 (6)	141
C31—H31*B*⋯O2^ii^	0.96	2.51	3.429 (7)	159
C31—H31*C*⋯O3^iii^	0.96	2.55	3.483 (7)	164

Symmetry codes: (i) 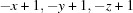; (ii) 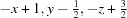; (iii) 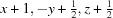.

**Table 3:** Experimental details

	(I)	(II)
Crystal data		
Chemical formula	[Co(C_16_H_14_N_2_O_2_)(NO_2_)(C_5_H_5_N)]	[Co(C_16_H_14_N_2_O_2_)(NO_2_)(C_6_H_7_N)]
*M*_r_	450.33	464.36
Crystal system, space group	Orthorhombic, *P*2_1_2_1_2_1_	Monoclinic, *P*2_1_/*c*
Temperature (K)	302	301
*a*, *b*, *c* (Å)	6.924 (2), 14.007 (3), 40.339 (8)	9.7430 (4), 18.0136 (6), 12.8488 (5)
α, β, γ (°)	90, 90, 90	90, 106.476 (1), 90
*V* (Å^3^)	3912.3 (16)	2162.45 (14)
*Z*	8	4
Radiation type	Mo *K*α	Mo *K*α
μ (mm^−1^)	0.91	0.83
Crystal size (mm)	0.29 × 0.06 × 0.04	0.30 × 0.10 × 0.07

Data collection		
Diffractometer	Bruker D8 VENTURE	Bruker D8 VENTURE
Absorption correction	Integration (*SADABS*; Bruker, 2016 ▸)	Integration (*SADABS*; Bruker, 2016 ▸)
*T*_min_, *T*_max_	0.841, 0.965	0.847, 0.952
No. of measured, independent and observed [*I* > 2σ(*I*)] reflections	52512, 9036, 6955	23719, 5114, 3793
*R*_int_	0.057	0.034
(sin θ/λ)_max_ (Å^−1^)	0.656	0.659

Refinement		
*R*[*F* ^2^ > 2σ(*F* ^2^)], *wR*(*F* ^2^), *S*	0.034, 0.081, 1.11	0.055, 0.192, 1.08
No. of reflections	9036	5114
No. of parameters	541	281
H-atom treatment	H-atom parameters constrained	H-atom parameters constrained
Δρ_max_, Δρ_min_ (e Å^−3^)	0.41, −0.38	1.25, −0.61
Absolute structure	Flack *x* determined using 2597 quotients [(*I* ^+^)−(*I* ^−^)]/[(*I* ^+^)+(*I* ^−^)] (Parsons *et al.*, 2013 ▸)	–
Absolute structure parameter	−0.010 (6)	–

Computer programs: *APEX3* and *SAINT* (Bruker, 2016 ▸), *SHELXT* (Sheldrick, 2015*a* ▸), *Mercury* (Macrae *et al.*, 2008 ▸), *CAVITY* (Ohashi *et al.*, 1981 ▸), *SHELXL2014* (Sheldrick, 2015*b* ▸) and *publCIF* (Westrip, 2010 ▸).

## supplementary crystallographic information

### trans-{2,2'-[Ethane-1,2-diylbis(nitrilomethylidyne)]diphenolato-κ4O,N,N',O'}(nitro-κN)(pyridine-κN)cobalt(III) (I) Crystal data

**Table:**

[Co(C_16_H_14_N_2_O_2_)(NO_2_)(C_5_H_5_N)]	*D*_x_ = 1.529 Mg m^−^^3^
*M**_r_* = 450.33	Mo *K*α radiation, λ = 0.71073 Å
Orthorhombic, *P*2_1_2_1_2_1_	Cell parameters from 9904 reflections
*a* = 6.924 (2) Å	θ = 2.5–26.4°
*b* = 14.007 (3) Å	µ = 0.91 mm^−^^1^
*c* = 40.339 (8) Å	*T* = 302 K
*V* = 3912.3 (16) Å^3^	Needle, brown
*Z* = 8	0.29 × 0.06 × 0.04 mm
*F*(000) = 1856	

### trans-{2,2'-[Ethane-1,2-diylbis(nitrilomethylidyne)]diphenolato-κ4O,N,N',O'}(nitro-κN)(pyridine-κN)cobalt(III) (I) Data collection

**Table:**

Bruker D8 VENTURE diffractometer	6955 reflections with *I* > 2σ(*I*)
φ and ω scans	*R*_int_ = 0.057
Absorption correction: integration (SADABS; Bruker, 2016)	θ_max_ = 27.8°, θ_min_ = 2.1°
*T*_min_ = 0.841, *T*_max_ = 0.965	*h* = −9→9
52512 measured reflections	*k* = −18→18
9036 independent reflections	*l* = −52→52

### trans-{2,2'-[Ethane-1,2-diylbis(nitrilomethylidyne)]diphenolato-κ4O,N,N',O'}(nitro-κN)(pyridine-κN)cobalt(III) (I) Refinement

**Table:**

Refinement on *F*^2^	Hydrogen site location: inferred from neighbouring sites
Least-squares matrix: full	H-atom parameters constrained
*R*[*F*^2^ > 2σ(*F*^2^)] = 0.034	*w* = 1/[σ^2^(*F*_o_^2^) + (0.0226*P*)^2^ + 1.3354*P*] where *P* = (*F*_o_^2^ + 2*F*_c_^2^)/3
*wR*(*F*^2^) = 0.081	(Δ/σ)_max_ = 0.001
*S* = 1.11	Δρ_max_ = 0.41 e Å^−^^3^
9036 reflections	Δρ_min_ = −0.38 e Å^−^^3^
541 parameters	Absolute structure: Flack *x* determined using 2597 quotients [(*I*^+^)-(*I*^-^)]/[(*I*^+^)+(*I*^-^)] (Parsons *et al.*, 2013)
0 restraints	Absolute structure parameter: −0.010 (6)
Primary atom site location: structure-invariant direct methods	

### trans-{2,2'-[Ethane-1,2-diylbis(nitrilomethylidyne)]diphenolato-κ4O,N,N',O'}(nitro-κN)(pyridine-κN)cobalt(III) (I) Special details

**Table:**

Geometry. All esds (except the esd in the dihedral angle between two l.s. planes) are estimated using the full covariance matrix. The cell esds are taken into account individually in the estimation of esds in distances, angles and torsion angles; correlations between esds in cell parameters are only used when they are defined by crystal symmetry. An approximate (isotropic) treatment of cell esds is used for estimating esds involving l.s. planes.

### trans-{2,2'-[Ethane-1,2-diylbis(nitrilomethylidyne)]diphenolato-κ4O,N,N',O'}(nitro-κN)(pyridine-κN)cobalt(III) (I) Fractional atomic coordinates and isotropic or equivalent isotropic displacement parameters (Å^2^)

**Table:**

	*x*	*y*	*z*	*U*_iso_*/*U*_eq_	
Co1	0.85121 (8)	0.53498 (3)	0.44179 (2)	0.02769 (12)	
Co2	0.35957 (8)	0.26710 (4)	0.28219 (2)	0.03077 (13)	
O3	0.6714 (6)	0.6081 (3)	0.49733 (8)	0.0730 (12)	
O4	0.7526 (7)	0.4641 (3)	0.50386 (8)	0.0762 (12)	
O5	0.6418 (4)	0.61244 (17)	0.42729 (6)	0.0339 (6)	
O6	0.6913 (4)	0.42812 (18)	0.43168 (6)	0.0330 (7)	
O7	0.2938 (7)	0.0765 (3)	0.26808 (10)	0.0924 (16)	
O8	0.1576 (7)	0.1673 (3)	0.23428 (10)	0.0816 (13)	
O9	0.1581 (4)	0.24890 (18)	0.31341 (6)	0.0352 (6)	
O10	0.1888 (4)	0.3519 (2)	0.25961 (6)	0.0384 (7)	
N11	0.7471 (5)	0.5353 (3)	0.48670 (8)	0.0372 (8)	
N12	1.0111 (5)	0.6392 (2)	0.45445 (8)	0.0340 (8)	
N13	1.0645 (5)	0.4574 (3)	0.45381 (7)	0.0327 (8)	
N14	0.9458 (5)	0.5370 (2)	0.39372 (7)	0.0311 (7)	
N15	0.2570 (5)	0.1572 (3)	0.25859 (9)	0.0396 (8)	
N16	0.5391 (5)	0.1846 (3)	0.30391 (9)	0.0389 (9)	
N17	0.5586 (5)	0.2833 (3)	0.25025 (8)	0.0392 (9)	
N18	0.4514 (5)	0.3825 (2)	0.30847 (8)	0.0352 (8)	
C19	0.6357 (7)	0.7062 (3)	0.42854 (9)	0.0347 (9)	
C20	0.4657 (7)	0.7517 (3)	0.41647 (10)	0.0453 (11)	
H20	0.3664	0.7149	0.4076	0.054*	
C21	0.4467 (9)	0.8500 (3)	0.41780 (12)	0.0565 (14)	
H21	0.3331	0.8783	0.4104	0.068*	
C22	0.5943 (9)	0.9073 (3)	0.43004 (12)	0.0588 (15)	
H22	0.5790	0.9732	0.4307	0.071*	
C23	0.7612 (8)	0.8667 (3)	0.44100 (11)	0.0485 (12)	
H23	0.8601	0.9057	0.4488	0.058*	
C24	0.7873 (6)	0.7656 (3)	0.44078 (10)	0.0370 (10)	
C25	0.9653 (7)	0.7287 (3)	0.45369 (9)	0.0396 (10)	
H25	1.0541	0.7722	0.4621	0.048*	
C26	1.1903 (6)	0.6075 (3)	0.47122 (10)	0.0426 (11)	
H26A	1.1685	0.6010	0.4949	0.051*	
H26B	1.2927	0.6537	0.4678	0.051*	
C27	1.2453 (6)	0.5121 (3)	0.45626 (11)	0.0420 (11)	
H27A	1.3024	0.5208	0.4345	0.050*	
H27B	1.3374	0.4792	0.4703	0.050*	
C28	1.0614 (7)	0.3662 (3)	0.45981 (9)	0.0369 (10)	
H28	1.1759	0.3376	0.4666	0.044*	
C29	0.8932 (7)	0.3063 (3)	0.45665 (9)	0.0350 (10)	
C30	0.9070 (8)	0.2098 (3)	0.46782 (10)	0.0443 (12)	
H30	1.0224	0.1879	0.4768	0.053*	
C31	0.7526 (9)	0.1493 (3)	0.46547 (11)	0.0524 (13)	
H31	0.7620	0.0871	0.4734	0.063*	
C32	0.5809 (8)	0.1813 (3)	0.45115 (11)	0.0497 (13)	
H32	0.4763	0.1399	0.4495	0.060*	
C33	0.5639 (7)	0.2740 (3)	0.43937 (10)	0.0398 (10)	
H33	0.4500	0.2931	0.4291	0.048*	
C34	0.7180 (6)	0.3401 (3)	0.44278 (10)	0.0333 (9)	
C35	0.9600 (6)	0.6196 (3)	0.37649 (10)	0.0377 (10)	
H35	0.9483	0.6771	0.3878	0.045*	
C36	0.9910 (7)	0.6218 (3)	0.34278 (10)	0.0438 (11)	
H36	0.9996	0.6801	0.3318	0.053*	
C37	1.0094 (6)	0.5382 (4)	0.32536 (10)	0.0452 (11)	
H37	1.0270	0.5387	0.3025	0.054*	
C38	1.0009 (6)	0.4531 (3)	0.34269 (10)	0.0407 (10)	
H38	1.0163	0.3952	0.3317	0.049*	
C39	0.9692 (6)	0.4549 (3)	0.37652 (10)	0.0357 (9)	
H39	0.9638	0.3973	0.3879	0.043*	
C40	0.1643 (7)	0.1934 (3)	0.33973 (9)	0.0341 (9)	
C41	0.0003 (7)	0.1897 (3)	0.36051 (10)	0.0413 (11)	
H41	−0.1075	0.2262	0.3552	0.050*	
C42	−0.0040 (9)	0.1333 (4)	0.38859 (11)	0.0534 (13)	
H42	−0.1152	0.1314	0.4015	0.064*	
C43	0.1563 (9)	0.0792 (3)	0.39774 (11)	0.0581 (13)	
H43	0.1535	0.0421	0.4169	0.070*	
C44	0.3176 (8)	0.0815 (3)	0.37825 (11)	0.0523 (13)	
H44	0.4237	0.0445	0.3841	0.063*	
C45	0.3277 (7)	0.1381 (3)	0.34951 (9)	0.0381 (10)	
C46	0.5049 (7)	0.1355 (3)	0.33027 (11)	0.0430 (11)	
H46	0.6021	0.0948	0.3376	0.052*	
C47	0.7144 (6)	0.1661 (4)	0.28403 (12)	0.0505 (12)	
H47A	0.6959	0.1102	0.2702	0.061*	
H47B	0.8242	0.1549	0.2985	0.061*	
C48	0.7488 (6)	0.2536 (4)	0.26278 (12)	0.0524 (13)	
H48A	0.8067	0.3042	0.2758	0.063*	
H48B	0.8346	0.2384	0.2445	0.063*	
C49	0.5353 (7)	0.3142 (3)	0.22032 (10)	0.0455 (11)	
H49	0.6417	0.3122	0.2062	0.055*	
C50	0.3591 (8)	0.3512 (3)	0.20717 (10)	0.0451 (10)	
C51	0.3481 (9)	0.3711 (4)	0.17269 (11)	0.0595 (13)	
H51	0.4541	0.3577	0.1593	0.071*	
C52	0.1864 (9)	0.4093 (4)	0.15869 (12)	0.0676 (17)	
H52	0.1793	0.4184	0.1359	0.081*	
C53	0.0304 (9)	0.4347 (4)	0.17923 (13)	0.0624 (15)	
H53	−0.0773	0.4642	0.1701	0.075*	
C54	0.0354 (7)	0.4162 (3)	0.21282 (11)	0.0495 (12)	
H54	−0.0684	0.4343	0.2260	0.059*	
C55	0.1962 (6)	0.3703 (3)	0.22745 (10)	0.0386 (11)	
C56	0.4742 (6)	0.4679 (3)	0.29333 (10)	0.0412 (10)	
H56	0.4693	0.4706	0.2703	0.049*	
C57	0.5047 (7)	0.5513 (3)	0.31087 (11)	0.0487 (12)	
H57	0.5201	0.6088	0.2997	0.058*	
C58	0.5122 (7)	0.5488 (4)	0.34477 (11)	0.0479 (12)	
H58	0.5288	0.6046	0.3569	0.058*	
C59	0.4945 (6)	0.4619 (4)	0.36050 (10)	0.0434 (11)	
H59	0.5031	0.4579	0.3835	0.052*	
C60	0.4639 (6)	0.3808 (3)	0.34170 (10)	0.0401 (10)	
H60	0.4515	0.3225	0.3525	0.048*	

### trans-{2,2'-[Ethane-1,2-diylbis(nitrilomethylidyne)]diphenolato-κ4O,N,N',O'}(nitro-κN)(pyridine-κN)cobalt(III) (I) Atomic displacement parameters (Å^2^)

**Table:**

	*U*^11^	*U*^22^	*U*^33^	*U*^12^	*U*^13^	*U*^23^
Co1	0.0270 (3)	0.0279 (3)	0.0282 (2)	−0.0004 (3)	−0.0004 (2)	−0.0005 (2)
Co2	0.0271 (3)	0.0352 (3)	0.0299 (3)	−0.0001 (3)	0.0000 (2)	−0.0016 (2)
O3	0.099 (3)	0.061 (2)	0.058 (2)	0.027 (2)	0.031 (2)	−0.0067 (17)
O4	0.124 (3)	0.055 (2)	0.0490 (19)	0.014 (2)	0.032 (2)	0.0148 (18)
O5	0.0319 (15)	0.0253 (13)	0.0445 (15)	0.0007 (14)	−0.0021 (14)	−0.0007 (11)
O6	0.0332 (17)	0.0265 (14)	0.0394 (15)	−0.0010 (12)	−0.0037 (12)	0.0017 (11)
O7	0.143 (4)	0.044 (2)	0.090 (3)	−0.018 (3)	−0.041 (3)	0.001 (2)
O8	0.087 (3)	0.071 (2)	0.087 (3)	0.013 (3)	−0.047 (3)	−0.034 (2)
O9	0.0356 (15)	0.0402 (16)	0.0299 (13)	0.0024 (15)	0.0034 (13)	0.0058 (12)
O10	0.0383 (18)	0.0445 (16)	0.0324 (14)	0.0029 (14)	−0.0005 (12)	0.0024 (13)
N11	0.0356 (19)	0.0391 (19)	0.0368 (18)	−0.0019 (19)	0.0010 (15)	−0.0020 (17)
N12	0.0295 (19)	0.039 (2)	0.0335 (17)	−0.0033 (16)	−0.0021 (15)	−0.0028 (15)
N13	0.0288 (18)	0.0395 (19)	0.0299 (16)	−0.0015 (16)	−0.0009 (14)	0.0012 (16)
N14	0.0311 (18)	0.0334 (18)	0.0289 (15)	−0.0008 (16)	−0.0019 (14)	−0.0001 (15)
N15	0.035 (2)	0.043 (2)	0.040 (2)	0.0008 (18)	0.0034 (17)	−0.0072 (18)
N16	0.030 (2)	0.040 (2)	0.046 (2)	0.0021 (17)	0.0002 (16)	0.0003 (17)
N17	0.032 (2)	0.050 (2)	0.0354 (18)	−0.0015 (18)	−0.0003 (16)	−0.0023 (17)
N18	0.0326 (19)	0.040 (2)	0.0334 (18)	−0.0018 (17)	−0.0033 (15)	0.0005 (15)
C19	0.043 (2)	0.030 (2)	0.0306 (19)	0.003 (2)	0.009 (2)	0.0021 (15)
C20	0.046 (3)	0.038 (3)	0.052 (3)	0.007 (2)	0.002 (2)	0.006 (2)
C21	0.074 (4)	0.040 (3)	0.055 (3)	0.023 (3)	0.002 (3)	0.005 (2)
C22	0.095 (5)	0.030 (2)	0.051 (3)	0.013 (3)	0.000 (3)	0.000 (2)
C23	0.074 (3)	0.033 (2)	0.039 (2)	−0.004 (2)	0.004 (2)	−0.004 (2)
C24	0.050 (3)	0.029 (2)	0.032 (2)	0.001 (2)	0.0057 (19)	−0.0049 (18)
C25	0.047 (3)	0.037 (2)	0.035 (2)	−0.013 (2)	0.0022 (19)	−0.0079 (19)
C26	0.035 (3)	0.054 (3)	0.038 (2)	−0.010 (2)	−0.0066 (19)	−0.002 (2)
C27	0.028 (2)	0.055 (3)	0.043 (2)	0.001 (2)	−0.0018 (19)	0.009 (2)
C28	0.038 (2)	0.042 (3)	0.031 (2)	0.012 (2)	−0.0025 (18)	−0.0004 (18)
C29	0.048 (3)	0.034 (2)	0.0234 (18)	0.003 (2)	0.0027 (18)	0.0009 (16)
C30	0.068 (4)	0.035 (2)	0.030 (2)	0.011 (2)	−0.004 (2)	0.0010 (18)
C31	0.094 (4)	0.028 (2)	0.036 (2)	0.000 (3)	0.008 (3)	0.0025 (19)
C32	0.072 (4)	0.033 (2)	0.044 (3)	−0.013 (2)	0.016 (2)	−0.007 (2)
C33	0.043 (2)	0.035 (2)	0.041 (2)	−0.006 (2)	0.007 (2)	−0.004 (2)
C34	0.039 (2)	0.030 (2)	0.0306 (19)	0.0006 (18)	0.0044 (18)	−0.0008 (18)
C35	0.042 (3)	0.035 (2)	0.037 (2)	−0.001 (2)	0.0011 (19)	0.0017 (19)
C36	0.047 (3)	0.047 (3)	0.037 (2)	−0.003 (2)	0.005 (2)	0.010 (2)
C37	0.042 (3)	0.061 (3)	0.033 (2)	0.008 (3)	0.0024 (19)	0.005 (2)
C38	0.040 (3)	0.044 (3)	0.038 (2)	0.004 (2)	0.0013 (19)	−0.012 (2)
C39	0.034 (2)	0.034 (2)	0.039 (2)	0.003 (2)	0.0006 (18)	−0.0036 (19)
C40	0.040 (3)	0.029 (2)	0.033 (2)	−0.004 (2)	0.004 (2)	−0.0044 (16)
C41	0.047 (3)	0.042 (3)	0.035 (2)	−0.004 (2)	0.005 (2)	−0.0027 (19)
C42	0.067 (4)	0.058 (3)	0.034 (2)	−0.014 (3)	0.013 (2)	−0.001 (2)
C43	0.082 (4)	0.057 (3)	0.035 (2)	−0.007 (3)	−0.003 (3)	0.014 (2)
C44	0.062 (4)	0.047 (3)	0.048 (3)	0.002 (3)	−0.006 (3)	0.011 (2)
C45	0.045 (3)	0.035 (2)	0.034 (2)	−0.004 (2)	−0.003 (2)	0.0020 (17)
C46	0.041 (3)	0.037 (2)	0.052 (3)	0.002 (2)	−0.011 (2)	0.005 (2)
C47	0.032 (2)	0.061 (3)	0.059 (3)	0.011 (2)	0.002 (2)	0.002 (3)
C48	0.027 (2)	0.075 (4)	0.055 (3)	−0.002 (3)	0.005 (2)	−0.001 (3)
C49	0.041 (3)	0.061 (3)	0.035 (2)	−0.008 (2)	0.007 (2)	−0.001 (2)
C50	0.044 (3)	0.056 (3)	0.036 (2)	−0.009 (3)	−0.001 (2)	0.003 (2)
C51	0.063 (3)	0.078 (4)	0.038 (2)	−0.015 (3)	0.005 (3)	0.008 (2)
C52	0.085 (5)	0.077 (4)	0.041 (3)	−0.015 (3)	−0.012 (3)	0.017 (3)
C53	0.068 (4)	0.063 (4)	0.056 (3)	−0.004 (3)	−0.021 (3)	0.019 (3)
C54	0.050 (3)	0.051 (3)	0.047 (3)	−0.005 (2)	−0.009 (2)	0.011 (2)
C55	0.043 (3)	0.040 (2)	0.032 (2)	−0.007 (2)	−0.0061 (18)	0.0035 (18)
C56	0.045 (3)	0.041 (2)	0.037 (2)	−0.009 (2)	0.0022 (19)	0.000 (2)
C57	0.049 (3)	0.041 (3)	0.056 (3)	−0.010 (2)	0.002 (2)	0.002 (2)
C58	0.040 (3)	0.051 (3)	0.053 (3)	−0.005 (2)	−0.003 (2)	−0.017 (2)
C59	0.038 (3)	0.057 (3)	0.035 (2)	0.002 (2)	−0.0058 (19)	−0.005 (2)
C60	0.039 (3)	0.045 (3)	0.036 (2)	−0.002 (2)	−0.0060 (19)	−0.001 (2)

### trans-{2,2'-[Ethane-1,2-diylbis(nitrilomethylidyne)]diphenolato-κ4O,N,N',O'}(nitro-κN)(pyridine-κN)cobalt(III) (I) Geometric parameters (Å, º)

**Table:**

Co1—N13	1.896 (3)	C30—H30	0.9300
Co1—N12	1.901 (3)	C31—C32	1.396 (7)
Co1—O5	1.903 (3)	C31—H31	0.9300
Co1—O6	1.906 (3)	C32—C33	1.389 (6)
Co1—N11	1.950 (3)	C32—H32	0.9300
Co1—N14	2.047 (3)	C33—C34	1.419 (6)
Co2—O9	1.897 (3)	C33—H33	0.9300
Co2—N17	1.900 (4)	C35—C36	1.377 (6)
Co2—O10	1.908 (3)	C35—H35	0.9300
Co2—N16	1.910 (4)	C36—C37	1.372 (6)
Co2—N15	1.944 (4)	C36—H36	0.9300
Co2—N18	2.035 (3)	C37—C38	1.384 (6)
O3—N11	1.224 (5)	C37—H37	0.9300
O4—N11	1.215 (4)	C38—C39	1.382 (5)
O5—C19	1.315 (4)	C38—H38	0.9300
O6—C34	1.325 (4)	C39—H39	0.9300
O7—N15	1.220 (5)	C40—C41	1.412 (6)
O8—N15	1.206 (5)	C40—C45	1.427 (6)
O9—C40	1.316 (4)	C41—C42	1.382 (6)
O10—C55	1.324 (5)	C41—H41	0.9300
N12—C25	1.295 (5)	C42—C43	1.394 (7)
N12—C26	1.481 (5)	C42—H42	0.9300
N13—C28	1.300 (5)	C43—C44	1.366 (7)
N13—C27	1.471 (5)	C43—H43	0.9300
N14—C39	1.353 (5)	C44—C45	1.407 (6)
N14—C35	1.353 (5)	C44—H44	0.9300
N16—C46	1.289 (5)	C45—C46	1.452 (6)
N16—C47	1.477 (5)	C46—H46	0.9300
N17—C49	1.293 (5)	C47—C48	1.514 (7)
N17—C48	1.471 (6)	C47—H47A	0.9700
N18—C60	1.343 (5)	C47—H47B	0.9700
N18—C56	1.352 (5)	C48—H48A	0.9700
C19—C20	1.424 (6)	C48—H48B	0.9700
C19—C24	1.428 (6)	C49—C50	1.427 (6)
C20—C21	1.385 (6)	C49—H49	0.9300
C20—H20	0.9300	C50—C55	1.419 (6)
C21—C22	1.389 (8)	C50—C51	1.421 (6)
C21—H21	0.9300	C51—C52	1.363 (7)
C22—C23	1.362 (7)	C51—H51	0.9300
C22—H22	0.9300	C52—C53	1.407 (8)
C23—C24	1.428 (6)	C52—H52	0.9300
C23—H23	0.9300	C53—C54	1.380 (7)
C24—C25	1.434 (6)	C53—H53	0.9300
C25—H25	0.9300	C54—C55	1.415 (6)
C26—C27	1.515 (6)	C54—H54	0.9300
C26—H26A	0.9700	C56—C57	1.382 (6)
C26—H26B	0.9700	C56—H56	0.9300
C27—H27A	0.9700	C57—C58	1.369 (6)
C27—H27B	0.9700	C57—H57	0.9300
C28—C29	1.441 (6)	C58—C59	1.378 (6)
C28—H28	0.9300	C58—H58	0.9300
C29—C34	1.417 (6)	C59—C60	1.382 (6)
C29—C30	1.428 (5)	C59—H59	0.9300
C30—C31	1.368 (7)	C60—H60	0.9300
			
N13—Co1—N12	85.27 (15)	C31—C30—H30	119.6
N13—Co1—O5	176.89 (12)	C29—C30—H30	119.6
N12—Co1—O5	95.08 (13)	C30—C31—C32	119.7 (4)
N13—Co1—O6	93.28 (13)	C30—C31—H31	120.2
N12—Co1—O6	176.70 (12)	C32—C31—H31	120.2
O5—Co1—O6	86.52 (12)	C33—C32—C31	121.0 (5)
N13—Co1—N11	92.96 (14)	C33—C32—H32	119.5
N12—Co1—N11	87.92 (14)	C31—C32—H32	119.5
O5—Co1—N11	90.14 (14)	C32—C33—C34	120.9 (4)
O6—Co1—N11	89.20 (13)	C32—C33—H33	119.6
N13—Co1—N14	90.07 (13)	C34—C33—H33	119.6
N12—Co1—N14	93.29 (14)	O6—C34—C29	124.3 (4)
O5—Co1—N14	86.83 (12)	O6—C34—C33	118.0 (4)
O6—Co1—N14	89.67 (13)	C29—C34—C33	117.7 (4)
N11—Co1—N14	176.83 (14)	N14—C35—C36	122.6 (4)
O9—Co2—N17	178.70 (14)	N14—C35—H35	118.7
O9—Co2—O10	86.83 (12)	C36—C35—H35	118.7
N17—Co2—O10	92.95 (14)	C37—C36—C35	120.0 (4)
O9—Co2—N16	95.33 (13)	C37—C36—H36	120.0
N17—Co2—N16	84.91 (16)	C35—C36—H36	120.0
O10—Co2—N16	177.68 (15)	C36—C37—C38	118.2 (4)
O9—Co2—N15	87.13 (14)	C36—C37—H37	120.9
N17—Co2—N15	91.59 (15)	C38—C37—H37	120.9
O10—Co2—N15	91.87 (15)	C39—C38—C37	119.3 (4)
N16—Co2—N15	89.08 (16)	C39—C38—H38	120.4
O9—Co2—N18	89.46 (13)	C37—C38—H38	120.4
N17—Co2—N18	91.81 (15)	N14—C39—C38	122.8 (4)
O10—Co2—N18	87.00 (13)	N14—C39—H39	118.6
N16—Co2—N18	92.18 (15)	C38—C39—H39	118.6
N15—Co2—N18	176.46 (15)	O9—C40—C41	118.3 (4)
C19—O5—Co1	125.6 (3)	O9—C40—C45	124.7 (4)
C34—O6—Co1	125.3 (3)	C41—C40—C45	117.0 (4)
C40—O9—Co2	126.2 (3)	C42—C41—C40	121.7 (5)
C55—O10—Co2	124.4 (3)	C42—C41—H41	119.2
O4—N11—O3	119.9 (3)	C40—C41—H41	119.2
O4—N11—Co1	121.0 (3)	C41—C42—C43	120.7 (5)
O3—N11—Co1	119.1 (3)	C41—C42—H42	119.6
C25—N12—C26	120.4 (4)	C43—C42—H42	119.6
C25—N12—Co1	126.5 (3)	C44—C43—C42	119.1 (4)
C26—N12—Co1	112.4 (3)	C44—C43—H43	120.5
C28—N13—C27	120.9 (4)	C42—C43—H43	120.5
C28—N13—Co1	126.7 (3)	C43—C44—C45	121.9 (5)
C27—N13—Co1	112.4 (3)	C43—C44—H44	119.1
C39—N14—C35	117.0 (3)	C45—C44—H44	119.1
C39—N14—Co1	120.8 (3)	C44—C45—C40	119.6 (4)
C35—N14—Co1	121.5 (3)	C44—C45—C46	117.9 (4)
O8—N15—O7	118.9 (4)	C40—C45—C46	122.4 (4)
O8—N15—Co2	120.9 (3)	N16—C46—C45	125.6 (4)
O7—N15—Co2	120.2 (3)	N16—C46—H46	117.2
C46—N16—C47	120.3 (4)	C45—C46—H46	117.2
C46—N16—Co2	125.6 (3)	N16—C47—C48	107.1 (4)
C47—N16—Co2	113.1 (3)	N16—C47—H47A	110.3
C49—N17—C48	121.8 (4)	C48—C47—H47A	110.3
C49—N17—Co2	125.6 (3)	N16—C47—H47B	110.3
C48—N17—Co2	112.5 (3)	C48—C47—H47B	110.3
C60—N18—C56	117.3 (4)	H47A—C47—H47B	108.5
C60—N18—Co2	121.7 (3)	N17—C48—C47	106.4 (4)
C56—N18—Co2	120.3 (3)	N17—C48—H48A	110.4
O5—C19—C20	117.4 (4)	C47—C48—H48A	110.4
O5—C19—C24	124.9 (4)	N17—C48—H48B	110.4
C20—C19—C24	117.7 (4)	C47—C48—H48B	110.4
C21—C20—C19	120.7 (5)	H48A—C48—H48B	108.6
C21—C20—H20	119.7	N17—C49—C50	125.1 (4)
C19—C20—H20	119.7	N17—C49—H49	117.5
C20—C21—C22	121.2 (5)	C50—C49—H49	117.5
C20—C21—H21	119.4	C55—C50—C51	119.0 (5)
C22—C21—H21	119.4	C55—C50—C49	122.3 (3)
C23—C22—C21	119.9 (4)	C51—C50—C49	118.7 (5)
C23—C22—H22	120.0	C52—C51—C50	121.8 (5)
C21—C22—H22	120.0	C52—C51—H51	119.1
C22—C23—C24	121.3 (5)	C50—C51—H51	119.1
C22—C23—H23	119.4	C51—C52—C53	119.0 (5)
C24—C23—H23	119.4	C51—C52—H52	120.5
C19—C24—C23	119.2 (4)	C53—C52—H52	120.5
C19—C24—C25	123.2 (4)	C54—C53—C52	120.8 (5)
C23—C24—C25	117.6 (4)	C54—C53—H53	119.6
N12—C25—C24	124.6 (4)	C52—C53—H53	119.6
N12—C25—H25	117.7	C53—C54—C55	121.0 (5)
C24—C25—H25	117.7	C53—C54—H54	119.5
N12—C26—C27	107.0 (3)	C55—C54—H54	119.5
N12—C26—H26A	110.3	O10—C55—C54	117.8 (4)
C27—C26—H26A	110.3	O10—C55—C50	124.0 (4)
N12—C26—H26B	110.3	C54—C55—C50	118.1 (4)
C27—C26—H26B	110.3	N18—C56—C57	122.3 (4)
H26A—C26—H26B	108.6	N18—C56—H56	118.8
N13—C27—C26	105.8 (3)	C57—C56—H56	118.8
N13—C27—H27A	110.6	C58—C57—C56	119.7 (4)
C26—C27—H27A	110.6	C58—C57—H57	120.2
N13—C27—H27B	110.6	C56—C57—H57	120.2
C26—C27—H27B	110.6	C57—C58—C59	118.6 (4)
H27A—C27—H27B	108.7	C57—C58—H58	120.7
N13—C28—C29	124.6 (4)	C59—C58—H58	120.7
N13—C28—H28	117.7	C58—C59—C60	119.1 (4)
C29—C28—H28	117.7	C58—C59—H59	120.4
C34—C29—C30	119.9 (4)	C60—C59—H59	120.4
C34—C29—C28	122.2 (4)	N18—C60—C59	122.9 (4)
C30—C29—C28	118.0 (4)	N18—C60—H60	118.6
C31—C30—C29	120.9 (5)	C59—C60—H60	118.6
			
O10—Co2—O9—C40	179.8 (3)	N14—C35—C36—C37	−0.2 (7)
N16—Co2—O9—C40	−1.0 (3)	C35—C36—C37—C38	−1.7 (7)
N15—Co2—O9—C40	87.8 (3)	C36—C37—C38—C39	1.7 (7)
N18—Co2—O9—C40	−93.1 (3)	C35—N14—C39—C38	−1.8 (6)
N12—Co1—N13—C28	162.7 (3)	Co1—N14—C39—C38	168.8 (3)
O6—Co1—N13—C28	−14.4 (3)	C37—C38—C39—N14	0.0 (7)
N11—Co1—N13—C28	75.0 (3)	Co2—O9—C40—C41	179.2 (3)
N14—Co1—N13—C28	−104.0 (3)	Co2—O9—C40—C45	1.1 (5)
N12—Co1—N13—C27	−16.8 (3)	O9—C40—C41—C42	−179.9 (4)
O6—Co1—N13—C27	166.1 (3)	C45—C40—C41—C42	−1.6 (6)
N11—Co1—N13—C27	−104.5 (3)	C40—C41—C42—C43	1.3 (7)
N14—Co1—N13—C27	76.5 (3)	C41—C42—C43—C44	−1.0 (8)
Co1—O5—C19—C20	−180.0 (3)	C42—C43—C44—C45	1.1 (8)
Co1—O5—C19—C24	0.6 (5)	C43—C44—C45—C40	−1.5 (7)
O5—C19—C20—C21	178.3 (4)	C43—C44—C45—C46	−179.7 (4)
C24—C19—C20—C21	−2.3 (6)	O9—C40—C45—C44	179.9 (4)
C19—C20—C21—C22	1.8 (7)	C41—C40—C45—C44	1.7 (6)
C20—C21—C22—C23	0.0 (7)	O9—C40—C45—C46	−2.0 (6)
C21—C22—C23—C24	−1.2 (7)	C41—C40—C45—C46	179.8 (4)
O5—C19—C24—C23	−179.4 (4)	C47—N16—C46—C45	−171.7 (4)
C20—C19—C24—C23	1.1 (6)	Co2—N16—C46—C45	−4.0 (6)
O5—C19—C24—C25	−0.8 (6)	C44—C45—C46—N16	−178.2 (4)
C20—C19—C24—C25	179.8 (4)	C40—C45—C46—N16	3.7 (7)
C22—C23—C24—C19	0.6 (6)	C46—N16—C47—C48	−160.9 (4)
C22—C23—C24—C25	−178.2 (4)	Co2—N16—C47—C48	29.9 (4)
C26—N12—C25—C24	−174.1 (4)	C49—N17—C48—C47	−140.7 (4)
Co1—N12—C25—C24	−4.6 (6)	Co2—N17—C48—C47	37.5 (5)
C19—C24—C25—N12	2.9 (6)	N16—C47—C48—N17	−41.9 (5)
C23—C24—C25—N12	−178.4 (4)	C48—N17—C49—C50	−174.9 (4)
C25—N12—C26—C27	−157.0 (4)	Co2—N17—C49—C50	7.1 (7)
Co1—N12—C26—C27	32.1 (4)	N17—C49—C50—C55	9.1 (8)
C28—N13—C27—C26	−141.8 (4)	N17—C49—C50—C51	−172.0 (4)
Co1—N13—C27—C26	37.8 (4)	C55—C50—C51—C52	1.0 (7)
N12—C26—C27—N13	−43.5 (4)	C49—C50—C51—C52	−177.9 (5)
C27—N13—C28—C29	−177.2 (4)	C50—C51—C52—C53	3.7 (8)
Co1—N13—C28—C29	3.3 (6)	C51—C52—C53—C54	−3.8 (8)
N13—C28—C29—C34	7.6 (6)	C52—C53—C54—C55	−0.8 (8)
N13—C28—C29—C30	−172.8 (4)	Co2—O10—C55—C54	165.5 (3)
C34—C29—C30—C31	−0.3 (6)	Co2—O10—C55—C50	−17.9 (6)
C28—C29—C30—C31	−179.9 (4)	C53—C54—C55—O10	−177.8 (4)
C29—C30—C31—C32	1.8 (6)	C53—C54—C55—C50	5.4 (7)
C30—C31—C32—C33	−0.3 (7)	C51—C50—C55—O10	178.0 (4)
C31—C32—C33—C34	−2.6 (6)	C49—C50—C55—O10	−3.2 (7)
Co1—O6—C34—C29	−16.9 (5)	C51—C50—C55—C54	−5.5 (6)
Co1—O6—C34—C33	165.7 (3)	C49—C50—C55—C54	173.4 (4)
C30—C29—C34—O6	−179.8 (4)	C60—N18—C56—C57	−1.6 (6)
C28—C29—C34—O6	−0.3 (6)	Co2—N18—C56—C57	169.0 (4)
C30—C29—C34—C33	−2.5 (6)	N18—C56—C57—C58	−0.1 (7)
C28—C29—C34—C33	177.1 (3)	C56—C57—C58—C59	1.9 (7)
C32—C33—C34—O6	−178.5 (4)	C57—C58—C59—C60	−2.0 (7)
C32—C33—C34—C29	3.9 (6)	C56—N18—C60—C59	1.4 (6)
C39—N14—C35—C36	1.9 (6)	Co2—N18—C60—C59	−169.0 (3)
Co1—N14—C35—C36	−168.7 (3)	C58—C59—C60—N18	0.4 (7)

### trans-{2,2'-[Ethane-1,2-diylbis(nitrilomethylidyne)]diphenolato-κ4O,N,N',O'}(nitro-κN)(pyridine-κN)cobalt(III) (I) Hydrogen-bond geometry (Å, º)

**Table:**

*D*—H···*A*	*D*—H	H···*A*	*D*···*A*	*D*—H···*A*
C25—H25···O3^i^	0.93	2.48	3.341 (5)	154
C38—H38···O9^ii^	0.93	2.39	3.280 (5)	160
C48—H48*B*···O8^ii^	0.97	2.48	3.285 (7)	140
C54—H54···O7^iii^	0.93	2.54	3.291 (7)	138
C59—H59···O6	0.93	2.38	3.213 (5)	149

Symmetry codes: (i) *x*+1/2, −*y*+3/2, −*z*+1; (ii) *x*+1, *y*, *z*; (iii) −*x*, *y*+1/2, −*z*+1/2.

### trans-{2,2'-[Ethane-1,2-diylbis(nitrilomethylidyne)]diphenolato-κ4O,N,N',O'}(4-methylpyridine-κN)(nitro-κN)cobalt(III) (II) Crystal data

**Table:**

[Co(C_16_H_14_N_2_O_2_)(NO_2_)(C_6_H_7_N)]	*F*(000) = 960
*M**_r_* = 464.36	*D*_x_ = 1.426 Mg m^−^^3^
Monoclinic, *P*2_1_/*c*	Mo *K*α radiation, λ = 0.71073 Å
*a* = 9.7430 (4) Å	Cell parameters from 9569 reflections
*b* = 18.0136 (6) Å	θ = 2.5–27.9°
*c* = 12.8488 (5) Å	µ = 0.83 mm^−^^1^
β = 106.476 (1)°	*T* = 301 K
*V* = 2162.45 (14) Å^3^	Needle, brown
*Z* = 4	0.30 × 0.10 × 0.07 mm

### trans-{2,2'-[Ethane-1,2-diylbis(nitrilomethylidyne)]diphenolato-κ4O,N,N',O'}(4-methylpyridine-κN)(nitro-κN)cobalt(III) (II) Data collection

**Table:**

Bruker D8 VENTURE diffractometer	3793 reflections with *I* > 2σ(*I*)
φ and ω scans	*R*_int_ = 0.034
Absorption correction: integration (SADABS; Bruker, 2016)	θ_max_ = 27.9°, θ_min_ = 2.0°
*T*_min_ = 0.847, *T*_max_ = 0.952	*h* = −11→12
23719 measured reflections	*k* = −23→23
5114 independent reflections	*l* = −16→16

### trans-{2,2'-[Ethane-1,2-diylbis(nitrilomethylidyne)]diphenolato-κ4O,N,N',O'}(4-methylpyridine-κN)(nitro-κN)cobalt(III) (II) Refinement

**Table:**

Refinement on *F*^2^	Primary atom site location: structure-invariant direct methods
Least-squares matrix: full	Hydrogen site location: inferred from neighbouring sites
*R*[*F*^2^ > 2σ(*F*^2^)] = 0.055	H-atom parameters constrained
*wR*(*F*^2^) = 0.192	*w* = 1/[σ^2^(*F*_o_^2^) + (0.0929*P*)^2^ + 3.2082*P*] where *P* = (*F*_o_^2^ + 2*F*_c_^2^)/3
*S* = 1.08	(Δ/σ)_max_ = 0.001
5114 reflections	Δρ_max_ = 1.25 e Å^−^^3^
281 parameters	Δρ_min_ = −0.61 e Å^−^^3^
0 restraints	

### trans-{2,2'-[Ethane-1,2-diylbis(nitrilomethylidyne)]diphenolato-κ4O,N,N',O'}(4-methylpyridine-κN)(nitro-κN)cobalt(III) (II) Special details

**Table:**

Geometry. All esds (except the esd in the dihedral angle between two l.s. planes) are estimated using the full covariance matrix. The cell esds are taken into account individually in the estimation of esds in distances, angles and torsion angles; correlations between esds in cell parameters are only used when they are defined by crystal symmetry. An approximate (isotropic) treatment of cell esds is used for estimating esds involving l.s. planes.

### trans-{2,2'-[Ethane-1,2-diylbis(nitrilomethylidyne)]diphenolato-κ4O,N,N',O'}(4-methylpyridine-κN)(nitro-κN)cobalt(III) (II) Fractional atomic coordinates and isotropic or equivalent isotropic displacement parameters (Å^2^)

**Table:**

	*x*	*y*	*z*	*U*_iso_*/*U*_eq_	
Co1	0.43909 (5)	0.31776 (2)	0.59728 (3)	0.03638 (18)	
O2	0.3197 (5)	0.4571 (2)	0.5824 (3)	0.0871 (12)	
O3	0.2071 (5)	0.3868 (2)	0.4603 (4)	0.1114 (17)	
O4	0.4759 (3)	0.35464 (13)	0.74031 (19)	0.0418 (6)	
O5	0.2919 (3)	0.26205 (15)	0.6288 (2)	0.0468 (6)	
N6	0.3024 (4)	0.39475 (19)	0.5412 (3)	0.0518 (8)	
N7	0.5772 (4)	0.37838 (17)	0.5612 (2)	0.0454 (7)	
N8	0.4123 (4)	0.27543 (18)	0.4586 (2)	0.0452 (7)	
N9	0.5822 (3)	0.23582 (16)	0.6577 (2)	0.0383 (6)	
C10	0.5620 (4)	0.40889 (18)	0.7823 (3)	0.0400 (8)	
C11	0.5769 (4)	0.4281 (2)	0.8917 (3)	0.0458 (9)	
H11	0.5257	0.4019	0.9308	0.055*	
C12	0.6657 (5)	0.4847 (2)	0.9408 (3)	0.0558 (10)	
H12	0.6746	0.4956	1.0132	0.067*	
C13	0.7428 (5)	0.5262 (2)	0.8859 (4)	0.0580 (11)	
H13	0.8014	0.5650	0.9201	0.070*	
C14	0.7303 (5)	0.5087 (2)	0.7802 (4)	0.0522 (10)	
H14	0.7808	0.5367	0.7424	0.063*	
C15	0.6436 (4)	0.44982 (19)	0.7263 (3)	0.0418 (8)	
C16	0.6455 (5)	0.4327 (2)	0.6194 (3)	0.0478 (9)	
H16	0.7003	0.4630	0.5882	0.057*	
C17	0.5788 (7)	0.3687 (3)	0.4473 (4)	0.0743 (15)	
H17A	0.5174	0.4053	0.4015	0.089*	
H17B	0.6752	0.3752	0.4416	0.089*	
C18	0.5273 (7)	0.2937 (3)	0.4122 (4)	0.0723 (14)	
H18A	0.4929	0.2918	0.3336	0.087*	
H18B	0.6048	0.2583	0.4362	0.087*	
C19	0.3099 (5)	0.2321 (2)	0.4085 (3)	0.0511 (10)	
H19	0.3095	0.2150	0.3401	0.061*	
C20	0.1964 (4)	0.2085 (2)	0.4515 (3)	0.0482 (9)	
C21	0.0852 (6)	0.1644 (3)	0.3846 (4)	0.0669 (14)	
H21	0.0896	0.1506	0.3159	0.080*	
C22	−0.0288 (6)	0.1418 (3)	0.4196 (5)	0.0764 (16)	
H22	−0.1002	0.1124	0.3752	0.092*	
C23	−0.0370 (5)	0.1626 (3)	0.5200 (5)	0.0748 (15)	
H23	−0.1159	0.1484	0.5425	0.090*	
C24	0.0683 (5)	0.2037 (3)	0.5877 (5)	0.0647 (12)	
H24	0.0605	0.2168	0.6558	0.078*	
C25	0.1902 (4)	0.2270 (2)	0.5558 (3)	0.0475 (9)	
C26	0.7169 (4)	0.2496 (2)	0.7154 (3)	0.0467 (9)	
H26	0.7466	0.2988	0.7266	0.056*	
C27	0.8141 (5)	0.1943 (2)	0.7592 (4)	0.0535 (10)	
H27	0.9066	0.2068	0.7992	0.064*	
C28	0.7751 (5)	0.1207 (2)	0.7443 (3)	0.0512 (9)	
C29	0.6333 (4)	0.1068 (2)	0.6881 (3)	0.0474 (9)	
H29	0.5996	0.0583	0.6790	0.057*	
C30	0.5427 (4)	0.1645 (2)	0.6460 (3)	0.0434 (8)	
H30	0.4489	0.1534	0.6072	0.052*	
C31	0.8785 (6)	0.0589 (3)	0.7867 (5)	0.0770 (15)	
H31A	0.9135	0.0400	0.7292	0.116*	
H31B	0.8310	0.0199	0.8139	0.116*	
H31C	0.9572	0.0772	0.8442	0.116*	

### trans-{2,2'-[Ethane-1,2-diylbis(nitrilomethylidyne)]diphenolato-κ4O,N,N',O'}(4-methylpyridine-κN)(nitro-κN)cobalt(III) (II) Atomic displacement parameters (Å^2^)

**Table:**

	*U*^11^	*U*^22^	*U*^33^	*U*^12^	*U*^13^	*U*^23^
Co1	0.0462 (3)	0.0347 (3)	0.0287 (3)	−0.00394 (18)	0.0115 (2)	−0.00221 (16)
O2	0.108 (3)	0.0533 (19)	0.093 (3)	0.023 (2)	0.017 (2)	−0.0042 (19)
O3	0.115 (4)	0.087 (3)	0.094 (3)	0.044 (3)	−0.033 (3)	−0.008 (2)
O4	0.0575 (16)	0.0381 (13)	0.0329 (11)	−0.0110 (11)	0.0176 (11)	−0.0052 (10)
O5	0.0464 (15)	0.0498 (15)	0.0444 (14)	−0.0112 (12)	0.0133 (12)	−0.0081 (11)
N6	0.059 (2)	0.0461 (18)	0.0486 (18)	0.0042 (15)	0.0132 (16)	−0.0021 (14)
N7	0.062 (2)	0.0422 (16)	0.0365 (15)	−0.0051 (14)	0.0220 (14)	−0.0011 (12)
N8	0.060 (2)	0.0472 (17)	0.0300 (14)	−0.0024 (15)	0.0151 (13)	−0.0021 (12)
N9	0.0462 (17)	0.0395 (15)	0.0306 (13)	−0.0046 (12)	0.0131 (12)	−0.0060 (11)
C10	0.051 (2)	0.0279 (15)	0.0373 (17)	0.0030 (14)	0.0066 (15)	−0.0005 (12)
C11	0.061 (2)	0.0393 (18)	0.0354 (17)	0.0030 (16)	0.0102 (16)	−0.0036 (14)
C12	0.068 (3)	0.050 (2)	0.043 (2)	0.005 (2)	0.0054 (19)	−0.0155 (17)
C13	0.059 (3)	0.040 (2)	0.063 (3)	−0.0035 (18)	−0.002 (2)	−0.0124 (18)
C14	0.051 (2)	0.0339 (18)	0.067 (3)	−0.0048 (16)	0.0082 (19)	−0.0005 (17)
C15	0.048 (2)	0.0313 (16)	0.0429 (18)	−0.0022 (14)	0.0082 (15)	0.0007 (14)
C16	0.060 (2)	0.0386 (18)	0.050 (2)	−0.0051 (17)	0.0229 (18)	0.0058 (16)
C17	0.114 (4)	0.073 (3)	0.051 (2)	−0.023 (3)	0.048 (3)	−0.009 (2)
C18	0.097 (4)	0.081 (3)	0.049 (2)	−0.016 (3)	0.036 (3)	−0.020 (2)
C19	0.071 (3)	0.0423 (19)	0.0305 (16)	0.0027 (18)	−0.0010 (17)	−0.0048 (14)
C20	0.051 (2)	0.0380 (18)	0.0448 (19)	−0.0009 (16)	−0.0049 (17)	0.0030 (15)
C21	0.077 (3)	0.045 (2)	0.054 (2)	−0.004 (2)	−0.020 (2)	0.0030 (19)
C22	0.062 (3)	0.053 (3)	0.089 (4)	−0.016 (2)	−0.020 (3)	0.010 (3)
C23	0.051 (3)	0.066 (3)	0.099 (4)	−0.017 (2)	0.007 (3)	0.005 (3)
C24	0.050 (3)	0.057 (3)	0.086 (3)	−0.009 (2)	0.018 (2)	−0.004 (2)
C25	0.043 (2)	0.0383 (18)	0.056 (2)	−0.0010 (15)	0.0057 (17)	−0.0003 (16)
C26	0.046 (2)	0.0418 (19)	0.052 (2)	−0.0082 (16)	0.0138 (17)	−0.0057 (16)
C27	0.043 (2)	0.055 (2)	0.061 (3)	−0.0071 (18)	0.0127 (19)	−0.0006 (19)
C28	0.055 (2)	0.048 (2)	0.051 (2)	0.0003 (18)	0.0162 (18)	0.0015 (17)
C29	0.058 (2)	0.0370 (18)	0.0455 (19)	−0.0040 (16)	0.0125 (17)	−0.0018 (15)
C30	0.050 (2)	0.0414 (18)	0.0364 (17)	−0.0061 (16)	0.0086 (15)	−0.0040 (14)
C31	0.060 (3)	0.065 (3)	0.104 (4)	0.014 (2)	0.019 (3)	0.014 (3)

### trans-{2,2'-[Ethane-1,2-diylbis(nitrilomethylidyne)]diphenolato-κ4O,N,N',O'}(4-methylpyridine-κN)(nitro-κN)cobalt(III) (II) Geometric parameters (Å, º)

**Table:**

Co1—O5	1.886 (3)	C17—H17A	0.9700
Co1—N8	1.887 (3)	C17—H17B	0.9700
Co1—O4	1.890 (2)	C18—H18A	0.9700
Co1—N7	1.891 (3)	C18—H18B	0.9700
Co1—N6	1.916 (4)	C19—C20	1.433 (6)
Co1—N9	2.027 (3)	C19—H19	0.9300
O2—N6	1.233 (5)	C20—C25	1.399 (6)
O3—N6	1.190 (5)	C20—C21	1.419 (6)
O4—C10	1.301 (4)	C21—C22	1.373 (8)
O5—C25	1.317 (4)	C21—H21	0.9300
N7—C16	1.295 (5)	C22—C23	1.367 (8)
N7—C17	1.478 (5)	C22—H22	0.9300
N8—C19	1.287 (5)	C23—C24	1.360 (7)
N8—C18	1.449 (6)	C23—H23	0.9300
N9—C26	1.335 (5)	C24—C25	1.426 (6)
N9—C30	1.338 (5)	C24—H24	0.9300
C10—C11	1.414 (5)	C26—C27	1.380 (6)
C10—C15	1.421 (5)	C26—H26	0.9300
C11—C12	1.371 (6)	C27—C28	1.378 (6)
C11—H11	0.9300	C27—H27	0.9300
C12—C13	1.387 (7)	C28—C29	1.388 (6)
C12—H12	0.9300	C28—C31	1.496 (6)
C13—C14	1.366 (6)	C29—C30	1.371 (6)
C13—H13	0.9300	C29—H29	0.9300
C14—C15	1.410 (5)	C30—H30	0.9300
C14—H14	0.9300	C31—H31A	0.9600
C15—C16	1.412 (5)	C31—H31B	0.9600
C16—H16	0.9300	C31—H31C	0.9600
C17—C18	1.465 (7)		
			
O5—Co1—N8	94.47 (13)	N7—C17—H17A	110.0
O5—Co1—O4	85.66 (11)	C18—C17—H17B	110.0
N8—Co1—O4	175.79 (13)	N7—C17—H17B	110.0
O5—Co1—N7	176.15 (13)	H17A—C17—H17B	108.4
N8—Co1—N7	85.35 (14)	N8—C18—C17	108.7 (4)
O4—Co1—N7	94.80 (12)	N8—C18—H18A	110.0
O5—Co1—N6	88.63 (14)	C17—C18—H18A	110.0
N8—Co1—N6	92.44 (15)	N8—C18—H18B	110.0
O4—Co1—N6	91.77 (13)	C17—C18—H18B	110.0
N7—Co1—N6	87.54 (15)	H18A—C18—H18B	108.3
O5—Co1—N9	90.72 (12)	N8—C19—C20	124.1 (3)
N8—Co1—N9	87.88 (13)	N8—C19—H19	117.9
O4—Co1—N9	87.91 (11)	C20—C19—H19	117.9
N7—Co1—N9	93.11 (13)	C25—C20—C21	118.8 (4)
N6—Co1—N9	179.30 (14)	C25—C20—C19	123.1 (3)
C10—O4—Co1	126.1 (2)	C21—C20—C19	118.2 (4)
C25—O5—Co1	124.5 (3)	C22—C21—C20	121.1 (5)
O3—N6—O2	117.5 (4)	C22—C21—H21	119.4
O3—N6—Co1	121.9 (3)	C20—C21—H21	119.4
O2—N6—Co1	120.2 (3)	C23—C22—C21	119.8 (5)
C16—N7—C17	120.9 (3)	C23—C22—H22	120.1
C16—N7—Co1	125.4 (3)	C21—C22—H22	120.1
C17—N7—Co1	112.5 (3)	C24—C23—C22	121.1 (5)
C19—N8—C18	120.8 (3)	C24—C23—H23	119.4
C19—N8—Co1	126.7 (3)	C22—C23—H23	119.4
C18—N8—Co1	112.4 (3)	C23—C24—C25	121.0 (5)
C26—N9—C30	116.6 (3)	C23—C24—H24	119.5
C26—N9—Co1	122.6 (2)	C25—C24—H24	119.5
C30—N9—Co1	120.8 (3)	O5—C25—C20	124.7 (4)
O4—C10—C11	117.9 (3)	O5—C25—C24	117.2 (4)
O4—C10—C15	124.6 (3)	C20—C25—C24	118.1 (4)
C11—C10—C15	117.6 (3)	N9—C26—C27	123.0 (4)
C12—C11—C10	120.9 (4)	N9—C26—H26	118.5
C12—C11—H11	119.6	C27—C26—H26	118.5
C10—C11—H11	119.6	C28—C27—C26	120.5 (4)
C11—C12—C13	121.9 (4)	C28—C27—H27	119.7
C11—C12—H12	119.0	C26—C27—H27	119.7
C13—C12—H12	119.0	C27—C28—C29	116.1 (4)
C14—C13—C12	118.3 (4)	C27—C28—C31	122.4 (4)
C14—C13—H13	120.9	C29—C28—C31	121.6 (4)
C12—C13—H13	120.9	C30—C29—C28	120.3 (4)
C13—C14—C15	122.3 (4)	C30—C29—H29	119.9
C13—C14—H14	118.9	C28—C29—H29	119.9
C15—C14—H14	118.9	N9—C30—C29	123.4 (4)
C14—C15—C16	118.1 (4)	N9—C30—H30	118.3
C14—C15—C10	119.0 (3)	C29—C30—H30	118.3
C16—C15—C10	122.9 (3)	C28—C31—H31A	109.5
N7—C16—C15	125.5 (3)	C28—C31—H31B	109.5
N7—C16—H16	117.3	H31A—C31—H31B	109.5
C15—C16—H16	117.3	C28—C31—H31C	109.5
C18—C17—N7	108.4 (4)	H31A—C31—H31C	109.5
C18—C17—H17A	110.0	H31B—C31—H31C	109.5
			
O5—Co1—O4—C10	−170.8 (3)	C17—N7—C16—C15	175.7 (4)
N7—Co1—O4—C10	5.4 (3)	Co1—N7—C16—C15	9.2 (6)
N6—Co1—O4—C10	−82.3 (3)	C14—C15—C16—N7	176.7 (4)
N9—Co1—O4—C10	98.3 (3)	C10—C15—C16—N7	−1.3 (6)
N8—Co1—O5—C25	−17.1 (3)	C16—N7—C17—C18	165.0 (5)
O4—Co1—O5—C25	167.1 (3)	Co1—N7—C17—C18	−26.9 (6)
N6—Co1—O5—C25	75.3 (3)	C19—N8—C18—C17	149.1 (4)
N9—Co1—O5—C25	−105.0 (3)	Co1—N8—C18—C17	−33.8 (6)
N8—Co1—N7—C16	174.4 (4)	N7—C17—C18—N8	38.1 (6)
O4—Co1—N7—C16	−9.8 (4)	C18—N8—C19—C20	176.8 (4)
N6—Co1—N7—C16	81.8 (4)	Co1—N8—C19—C20	0.1 (6)
N9—Co1—N7—C16	−98.0 (3)	N8—C19—C20—C25	−3.8 (6)
N8—Co1—N7—C17	6.9 (3)	N8—C19—C20—C21	176.0 (4)
O4—Co1—N7—C17	−177.3 (3)	C25—C20—C21—C22	1.8 (6)
N6—Co1—N7—C17	−85.7 (3)	C19—C20—C21—C22	−178.1 (4)
N9—Co1—N7—C17	94.5 (3)	C20—C21—C22—C23	0.8 (7)
O5—Co1—N8—C19	8.3 (3)	C21—C22—C23—C24	−1.9 (8)
N7—Co1—N8—C19	−167.8 (4)	C22—C23—C24—C25	0.5 (8)
N6—Co1—N8—C19	−80.5 (3)	Co1—O5—C25—C20	18.4 (5)
N9—Co1—N8—C19	98.9 (3)	Co1—O5—C25—C24	−164.4 (3)
O5—Co1—N8—C18	−168.7 (3)	C21—C20—C25—O5	174.0 (4)
N7—Co1—N8—C18	15.2 (3)	C19—C20—C25—O5	−6.1 (6)
N6—Co1—N8—C18	102.5 (4)	C21—C20—C25—C24	−3.1 (6)
N9—Co1—N8—C18	−78.1 (3)	C19—C20—C25—C24	176.7 (4)
Co1—O4—C10—C11	−179.3 (3)	C23—C24—C25—O5	−175.3 (4)
Co1—O4—C10—C15	0.0 (5)	C23—C24—C25—C20	2.1 (7)
O4—C10—C11—C12	−179.9 (4)	C30—N9—C26—C27	1.8 (6)
C15—C10—C11—C12	0.8 (6)	Co1—N9—C26—C27	178.3 (3)
C10—C11—C12—C13	1.0 (6)	N9—C26—C27—C28	0.3 (7)
C11—C12—C13—C14	−1.0 (7)	C26—C27—C28—C29	−2.9 (6)
C12—C13—C14—C15	−0.7 (6)	C26—C27—C28—C31	177.6 (4)
C13—C14—C15—C16	−175.6 (4)	C27—C28—C29—C30	3.4 (6)
C13—C14—C15—C10	2.4 (6)	C31—C28—C29—C30	−177.0 (4)
O4—C10—C15—C14	178.4 (3)	C26—N9—C30—C29	−1.2 (5)
C11—C10—C15—C14	−2.4 (5)	Co1—N9—C30—C29	−177.8 (3)
O4—C10—C15—C16	−3.7 (6)	C28—C29—C30—N9	−1.5 (6)
C11—C10—C15—C16	175.6 (4)		

### trans-{2,2'-[Ethane-1,2-diylbis(nitrilomethylidyne)]diphenolato-κ4O,N,N',O'}(4-methylpyridine-κN)(nitro-κN)cobalt(III) (II) Hydrogen-bond geometry (Å, º)

**Table:**

*D*—H···*A*	*D*—H	H···*A*	*D*···*A*	*D*—H···*A*
C16—H16···O2^i^	0.93	2.58	3.358 (6)	141
C31—H31*B*···O2^ii^	0.96	2.51	3.429 (7)	159
C31—H31*C*···O3^iii^	0.96	2.55	3.483 (7)	164

Symmetry codes: (i) −*x*+1, −*y*+1, −*z*+1; (ii) −*x*+1, *y*−1/2, −*z*+3/2; (iii) *x*+1, −*y*+1/2, *z*+1/2.
