# Supplementary figures and images for: Investigation of nitro–nitrito photoisomerization: crystal structures of trans-{2,2′-[ethane-1,2-diylbis(nitrilo­methyl­idyne)]diphenolato}(pyridine/4-methyl­pyridine)­nitro­cobalt(III)
Source: Acta Crystallogr E Crystallogr Commun. 2018 Nov 9;74(Pt 12):1759–63. doi: 10.1107/S2056989018015487 (PMC6281106; doi:10.1107/S2056989018015487)

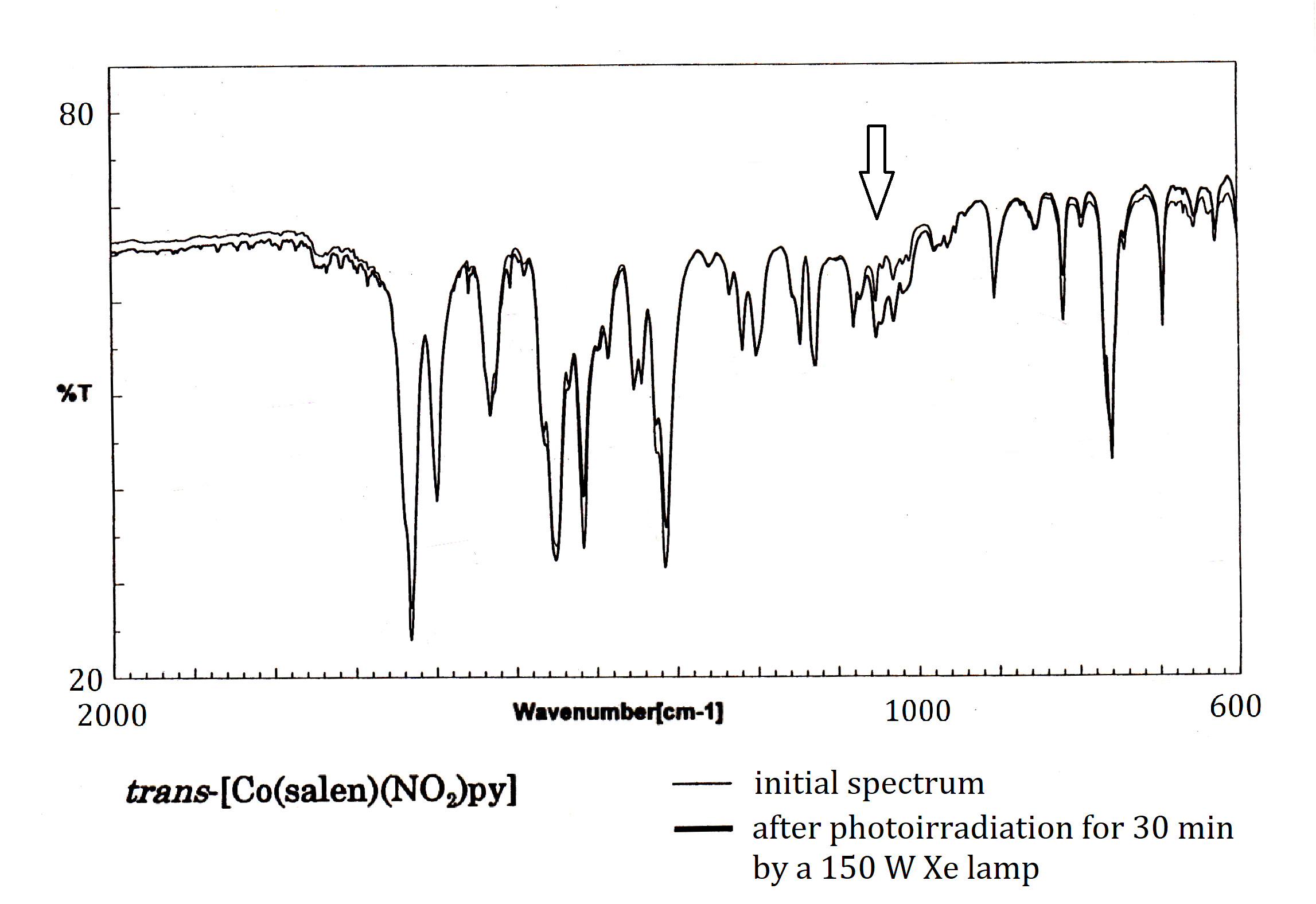

Supplement: Supplementary file 6 [file e-74-01759-sup6.tif]
